# Wearable Augmented Reality for Dental Implant Navigation: From Experimental Models to Clinical Application—A Scoping Review

**DOI:** 10.3390/dj14080518

**Published:** 2026-08-13

**Authors:** Angelo Auricchio, Antonio Lanza, Roberto Duraccio

**Affiliations:** 1 Department of Medicine, Surgery and Dentistry “Scuola Medica Salernitana”, DIPMED, University of Salerno, Via S. Allende, 84084 Baronissi, Italy; alanza@unisa.it; 2 Private Practice, Via Roma 72, 80047 San Giuseppe Vesuviano, Italy; odontoiatriaduraccio@gmail.com

**Keywords:** augmented reality, dental implantology, dynamic navigation, head-mounted display, dental implantation accuracy, scoping review

## Abstract

**Background:** Accurate three-dimensional positioning of dental implants is fundamental for long-term prosthetic success. While static guided surgery and traditional dynamic navigation present intrinsic limitations related to physical bulk or hand–eye coordination (the “look-away” phenomenon), Augmented Reality (AR) via Head-Mounted Displays (HMDs) promises to overcome these barriers by superimposing virtual holograms directly onto the surgical field. **Objectives:** The aim of this scoping review is to map the existing literature to evaluate the positioning accuracy, workflow efficiency, and ergonomic/technical barriers of HMD-based AR navigation systems in implant dentistry. **Methods:** A systematic search was conducted across the PubMed, Scopus, Web of Science, and Cochrane Library databases, following the PRISMA-ScR guidelines. In vitro, ex vivo, and clinical studies utilizing AR headsets (e.g., HoloLens) for implant guidance, reporting quantitative data on accuracy and/or qualitative data on usability issues, were included. **Results:** Eleven studies were included (7 in vitro, 1 ex vivo, 3 clinical). Accuracy showed a strong dependence on case complexity: for standard implants, the mean error at the entry point ranged between 0.33 and 1.51 mm, which is comparable to s-CAIS techniques. However, in complex scenarios (zygomatic implants), significant deviations were reported (up to 5.64 mm and 9.60°). The main barriers to routine adoption identified include tracking instability due to line-of-sight interruption, device weight, and the vergence–accommodation conflict. **Conclusions:** AR dynamic navigation demonstrates promising potential. Nevertheless, current evidence relies predominantly on in vitro studies that fail to fully replicate real-world surgical complexities. Persistent technical and ergonomic barriers, such as tracking instability and hardware limitations, indicate that the technology remains largely in a preclinical phase and is not yet suitable for routine clinical application. Further advancements toward markerless tracking systems, lighter headsets, and rigorous in vivo trials are required.

## 1. Introduction

### 1.1. The Clinical Context

Implant therapy currently represents a reliable and predictable solution for the rehabilitation of edentulism, with reported 5-year survival rates exceeding 95% for implant-supported single crowns, although it is not exempt from technical and biological complications [1]. However, long-term success and the stability of peri-implant tissues do not depend solely on osseointegration but are intrinsically linked to the correct three-dimensional positioning of the implant, a crucial factor for preserving the buccal bone wall and ensuring optimal aesthetic results over time [2]. The evolution of digital technologies has consolidated the importance of virtual planning in improving treatment predictability compared to conventional procedures [3]. Nevertheless, free-hand surgical execution remains subject to human variables related to the operator’s experience, entailing risks of deviation between the planned and actual implant position [4].

### 1.2. The Evolution of Guided Surgery

To mitigate the uncertainties related to manual execution and faithfully transfer the virtual plan to clinical reality, Computer-Assisted Implant Surgery (CAIS) was introduced, categorized into static (s-CAIS) and dynamic (d-CAIS) [5]. Static guided surgery currently represents the most widespread and documented digital method: utilizing stereolithographic templates that physically constrain the direction of the drills, this technique has been shown to significantly reduce positioning error compared to dental navigation, with a reported mean error at the entry point of approximately 1.2 mm and at the apex of 1.4 mm [6,7]. However, despite its high accuracy, s-CAIS presents intrinsic limitations that reduce its versatility: the physical bulk of the template can hinder irrigation of the osteotomy site and direct vision, production costs remain high, and, critically, the “static” nature of the system prevents the surgeon from modifying the treatment plan intraoperatively in response to unforeseen anatomical conditions [8].

Dynamic navigation (d-CAIS) has emerged as a flexible alternative, utilizing optical tracking systems to monitor the position of surgical instruments in real-time relative to the patient’s anatomy, eliminating the need for physical guides; Block et al. (2016) stated that as the experience of the clinician and their surgical proficiency increases, the use of the dynamic method might predominate, because of the time- and cost-efficient workflow [5,9]. Although this technology resolves the issues of bulk and cooling typical of templates, traditional systems based on external monitors introduce a complex ergonomic barrier known as the “look-away” phenomenon. The surgeon is forced to avert their gaze from the surgical field to view the navigation screen, interrupting natural hand–eye coordination and imposing a steep learning curve to manage the cognitive dissociation between manual action and remote visual feedback [10].

### 1.3. The Digital Evolution, the Virtuality Continuum, and the Advent of Augmented Reality

The digital revolution has radically transformed modern dentistry through the widespread adoption of Cone Beam Computed Tomography (CBCT), intraoral scanners (IOS), and Computer-Aided Design (CAD) software. Within this digital framework, Augmented Reality (AR) has emerged as a highly promising technology [11,12]. To properly understand its role, it is necessary to refer to the “Virtuality Continuum” taxonomy introduced by Milgram and Kishino in 1994. According to this framework, visual display environments are classified along a continuous scale with “completely real environments” at one extremum and “completely virtual environments” (Virtual Reality, VR) at the opposite. Mixed Reality (MR) is defined as any hybrid environment that blends real and virtual worlds somewhere along this continuum. Augmented Reality represents a specific subset of MR situated closer to the real-world extremum; it refers to cases where an otherwise completely real environment is “augmented” by the superposition of computer-generated virtual objects, whereas Augmented Virtuality (AV) describes a primarily virtual environment enhanced by real-world imagery [13]. Unlike VR, which completely immerses and isolates the user in a synthetic environment, AR allows clinicians to seamlessly interact with both physical anatomy and digital holograms simultaneously. Consequently, AR is currently being investigated across various dental fields, including oral and maxillofacial surgery (OMS), restorative dentistry, orthodontics, endodontics, and preclinical academic education [14,15,16].

In an innovative proof-of-concept protocol, Mangano et al. have demonstrated the feasibility of authentic three-dimensional (3D) implant planning, which allows clinicians to bypass traditional 2D radiological software for guided surgery. In this workflow, AI is initially employed for the automatic alignment and rapid segmentation of data derived from cone-beam computed tomography (CBCT) and intraoral scans [17].

In the specific context of surgical navigation, AR was introduced to solve the intrinsic ergonomic limitations of traditional systems, often described in the literature as the “Data Availability Paradox” [18]. During complex procedures, the sheer volume of intraoperative data presented on external monitors can easily lead to sensory overload and cognitive fatigue. Furthermore, relying on conventional computer-guided dynamic navigation requires the surgeon to frequently look away from the surgical site to check the display, a distraction that increases the risk of accidental instrument displacement [10,18,19].

By utilizing Head-Mounted Displays (HMDs), AR provides a direct “egocentric” visualization, projecting the planned holograms perfectly focused within the surgical field alongside the real-world image, allowing the surgeon to maintain uninterrupted visual and cognitive focus on the patient [19,20]. Moreover, AR functionally bypasses the final limiting step of the conventional digital dentistry workflow: instead of transferring a digital plan back into a solid physical state (such as 3D-printing a static surgical guide), the digital data is directly visualized onto the patient. This direct holographic visualization significantly reduces workflow time, prevents potential data loss during physical manufacturing, and offers an authentic three-dimensional planning and operative environment [15,18].

### 1.4. Augmented Reality in Implant Dentistry

In the field of implant dentistry, Augmented Reality (AR) represents an advanced tool designed to bridge the gap between three-dimensional virtual planning and the actual surgical procedure [11,14,15,21]. By utilizing wearable displays, AR overlays digital information—such as the planned implant trajectory and critical anatomical boundaries like the inferior alveolar nerve or the maxillary sinus—directly onto the surgical field. Unlike static Computer-Aided Implant Surgery (s-CAIS), which relies on physical surgical guides (e.g., tooth-supported templates) that can obstruct direct visual feedback and limit irrigation, AR provides dynamic visual guidance without physically hindering the operative site [15,20]. A comprehensive systematic review and meta-analysis by Mai et al. (2023) concluded that AR-DN provides accuracy analogous to s-CAIS and is significantly more precise than both traditional FH positioning and conventional dynamic navigation [22].

Current Limitations: Despite these promising ergonomic and visual advantages, an objective evaluation of the current literature indicates that AR technology in implantology is still in a developmental phase. Most of the available evidence is currently derived from in vitro experiments or preclinical models, where the controlled environment strongly favors optimal device performance [15,19]. In a dynamic real-world clinical setting, several technological challenges persist, including tracking errors, system latency, and the difficulty of consistently maintaining the sub-millimeter accuracy required for safe implant placement [10]. Consequently, while AR holds immense potential to enhance surgical navigation, claiming full clinical maturity is currently premature. Further high-quality in vivo studies are necessary to fully validate its accuracy and reliability before it can be considered a routine alternative to conventional guided surgery workflows [10,17,23,24].

### 1.5. Technological Advancement and Clinical Application Gap

Despite the transformative potential of this technology, the adoption of AR in the daily surgical workflow remains limited, and the scientific literature still appears fragmented. A systematic review of current applications reveals that much of the research focuses predominantly on the use of AR as a tool for pre-clinical education and simulated student training, exploiting the ability of headsets to display interactive anatomical models to improve the learning curve without risk to the patient [25,26]. In contrast, studies investigating active intraoperative application are numerically fewer and mostly confined to in vitro experiments on ideal models or phantoms, which do not replicate the environmental complexities of the real operating room, such as soft tissue management, the presence of saliva, or the patient’s involuntary movements [27]. Even more scarce is the amount of clinical evidence on real patients (“in vivo”), which leaves critical questions open regarding the feasibility and reliability of the system in uncontrolled scenarios [28]. Furthermore, while accuracy seems promising for standard cases, recent comparative investigations have raised significant doubts about the performance of AR in complex rehabilitations, such as zygomatic implants, where the length of the drilling path amplifies angular deviations and requires tracking stability that current hardware may not fully guarantee [29,30].

This gap between theoretical expectations and clinical reality suggests the presence of technical barriers and ergonomic barriers that are not yet fully codified [23], making a systematic analysis necessary to map the state of the art and define the true Technology Readiness Level of AR in implant dentistry.

### 1.6. Aim of the Study

Considering these premises, the present work aims to conduct a scoping review of the existing literature to systematically map the state of the art regarding the application of dynamic navigation systems based on Augmented Reality with wearable displays (HMDs) in implant dentistry. Specifically, the review intends to: (1) synthesize quantitative data regarding implant positioning accuracy (entry point, apex, and angular deviations), analyzing the differences between standard and complex scenarios; (2) identify and categorize the technical and ergonomic barriers, such as tracking instability and operator discomfort, that currently hinder its routine adoption; and (3) evaluate the true clinical feasibility of this technology compared to conventional static or free-hand methods, in order to guide future research and development directions.

## 2. Materials and Methods

This scoping review was conducted following the methodological guidelines of the Joanna Briggs Institute (JBI) for Scoping Reviews. A formal review protocol was not registered prior to the commencement of this study. However, the search strategy and the drafting of the manuscript are strictly in accordance with the PRISMA-ScR (Preferred Reporting Items for Systematic reviews and Meta-Analyses extension for Scoping Reviews) checklist [31]. The systematic search of the literature was performed between November 2025 and February 2026.

### 2.1. Eligibility Criteria

To define the inclusion and exclusion criteria, the PCC (Population, Concept, Context) framework was adopted:

**Population:** Studies regarding the placement of dental implants (standard or zygomatic) performed both on real patients (in vivo) and on anatomical models or phantom heads (in vitro/ex vivo) were included. Studies on non-standardized animal models were excluded.

**Concept:** The intervention under investigation is Dynamic Navigation in Augmented Reality (AR) supported exclusively by wearable devices (Head-Mounted Displays—HMDs or Smart Glasses), such as Microsoft HoloLens (Microsoft Corporation, Redmond, WA, USA) or Epson Moverio (Seiko Epson Corporation, Suwa, Japan). Fundamental Exclusion Criteria: Studies using AR solely for passive visualization in combination with static surgical templates (Static Guides), robot-assisted surgery (where the robotic arm performs the drilling), studies evaluating interventions outside the strict scope of dental implant placement (such as orthodontic miniscrew insertion, extra-oral surgeries, or AR guidance for fixed prosthodontics) were explicitly excluded due to incorrect intervention or context. Non-wearable AR systems (tablets, smartphones, external mirror monitors) were excluded. Furthermore, to maintain a strict focus on technical performance (positioning accuracy) and practical ergonomics during active implant surgery, secondary literature such as narrative reviews, systematic reviews, and other scoping reviews were explicitly excluded. The primary objective was to extract direct, raw data from primary studies (in vitro, ex vivo, and clinical) where AR headsets were actively and physically utilized for surgical guidance.

**Context:** Studies reporting quantitative accuracy data (entry point, apex, and angular deviations) and/or qualitative/quantitative data related to workflow, ergonomics, and technical barriers (e.g., setup time, tracking issues, visual fatigue) were considered. Editorials, expert opinions, and purely engineering/algorithmic studies lacking clinical or pre-clinical validation on dental anatomy were excluded.

### 2.2. Information Sources and Search Strategy

A systematic literature search was performed across the following electronic databases: PubMed (MEDLINE), Scopus, Web of Science (WoS), and the Cochrane Library. The search strategy combined keywords and MeSH terms related to three main domains:

Technology (“Augmented Reality”, “Mixed Reality”, “Head-Mounted Display”, “HoloLens”, “Smart Glasses”);

Clinical field (“Dental Implants”, “Implantology”, “Oral Surgery”);

Outcome (“Navigation”, “Accuracy”, “Guided Surgery”).

Full search strategy strings are available in the “Data Availability Statement” section.

No strict retroactive time limits were applied; however, the search was updated to include all studies published up to February 2026, marking the completion of the manuscript drafting. The reference lists of the included studies were manually screened to identify further relevant articles.

### 2.3. Selection of Sources of Evidence

The identified records were imported into reference management softwares: Zotero V9.0.4 (Corporation for Digital Scholarship, Vienna, VA, USA) and Mendeley V2.142.0 (Elsevier, Amsterdam, The Netherlands), duplicates were removed. The selection process was conducted blindly by two investigators (A.A. and R.D.), the overall alignment was 88%; any disagreements were resolved by a third investigator (A.L.). The process was carried out in two phases:

Initial Screening: Evaluation of titles and abstracts to exclude clearly irrelevant articles (e.g., reviews, wrong context/interventions, AR HMDs in education/training, non-HMDs AR navigation, engineering studies).

Full-Text Assessment: Full-text reading of potentially eligible articles to verify compliance with the inclusion/exclusion criteria defined in the PCC framework. The reasons for exclusion at this stage (e.g., “Wrong Intervention”, “Wrong Outcome”) were recorded and reported in the PRISMA-ScR flow diagram.

### 2.4. Data Charting Process

Data from the included studies were extracted using a standardized data collection form (spreadsheet), specifically developed for the objectives of this review. The extracted variables include:

Study Characteristics: Author, year of publication, study design (in vitro, in vivo, Case series, RCT).

Technology: Type of HMD device used (e.g., HoloLens 2 Microsoft Corporation, Redmond, WA, USA; Epson Moverio Seiko Epson Corporation, Suwa, Japan), tracking method (optical with markers, markerless, infrared).

Clinical Scenario: Type of edentulism (single, partial, total) and implant typology (standard vs. zygomatic).

Accuracy Outcomes: Mean and Standard Deviation (SD) of the error at the entry point (mm), at the apex (mm), and angular deviation (degrees).

Ergonomic and Technical Outcomes: Reported data on setup times, “line-of-sight” issues, device comfort, visual fatigue (Vergence–Accommodation Conflict), and system stability.

The extracted data have been formalized and summarized in Table 1.

### 2.5. Critical Appraisal of Individual Sources of Evidence

In accordance with the PRISMA-ScR guidelines, a formal risk of bias assessment or critical appraisal of the included sources of evidence was not conducted. The primary objective of this scoping review is to broadly map the existing literature and identify technological and ergonomic barriers, rather than to evaluate the methodological quality or the weight of the evidence of the individual studies.

### 2.6. Synthesis of Results

Due to the heterogeneity of the study designs (the majority being in vitro and case series) and the different devices used, it was not possible to conduct a statistical meta-analysis. The results were synthesized in narrative and tabular form. The analysis was structured into three main thematic areas:

Characteristics Analysis: Overview of the predominant devices and tracking methods.

Quantitative Synthesis of Accuracy: Comparison of the reported error ranges, distinguishing between standard cases and complex cases (zygomatic).

Barriers Analysis: Thematic mapping of the technical (hardware, software) and ergonomic limitations that hinder routine clinical adoption.

## 3. Results

### 3.1. Study Selection

Although the literature search was continuously updated to include records published up to February 2026, the final pool of included articles ranges strictly from 2019 to 2024.

The initial systematic search identified a total of 249 records across the electronic databases (PubMed, Scopus, Web of Science, and Cochrane Library). After the removal of 102 duplicates, 147 records underwent preliminary screening based on title and abstract. Of these, 121 were excluded as they were not relevant to the research topic. The screening was conducted using Rayyan platform by two blinded reviewers (A.A. and R.D.) with an alignment of 88% (19 discordant articles); in case of difficulties in resolution, a third reviewer (A.L.) was consulted. The main reason for the discrepancy between the two primary investigators was difficulty in interpreting the context of application, the type of technology used (VR vs. AR) and the type of surgery performed. Twenty-six reports were sought for full-text retrieval; however, three could not be retrieved. The remaining 23 full-text articles were assessed for eligibility according to the pre-established inclusion and exclusion criteria.

Following the full-text review, 12 studies were excluded. The main reasons for exclusion were: wrong intervention (e.g., robotic surgery or non-HMD navigation systems) (*n* = 4), wrong context (e.g., purely engineering or algorithmic focus) (*n* = 3), descriptive only (design/planning focus) (*n* = 3), and unsuitable setting/subjects (e.g., studies on non-standardized animal models or obsolete technologies) (*n* = 2). During the full-text assessment phase, several recent articles published in 2025 and early 2026 were evaluated but ultimately excluded as they did not meet the stringent eligibility criteria.

**Figure 1:**
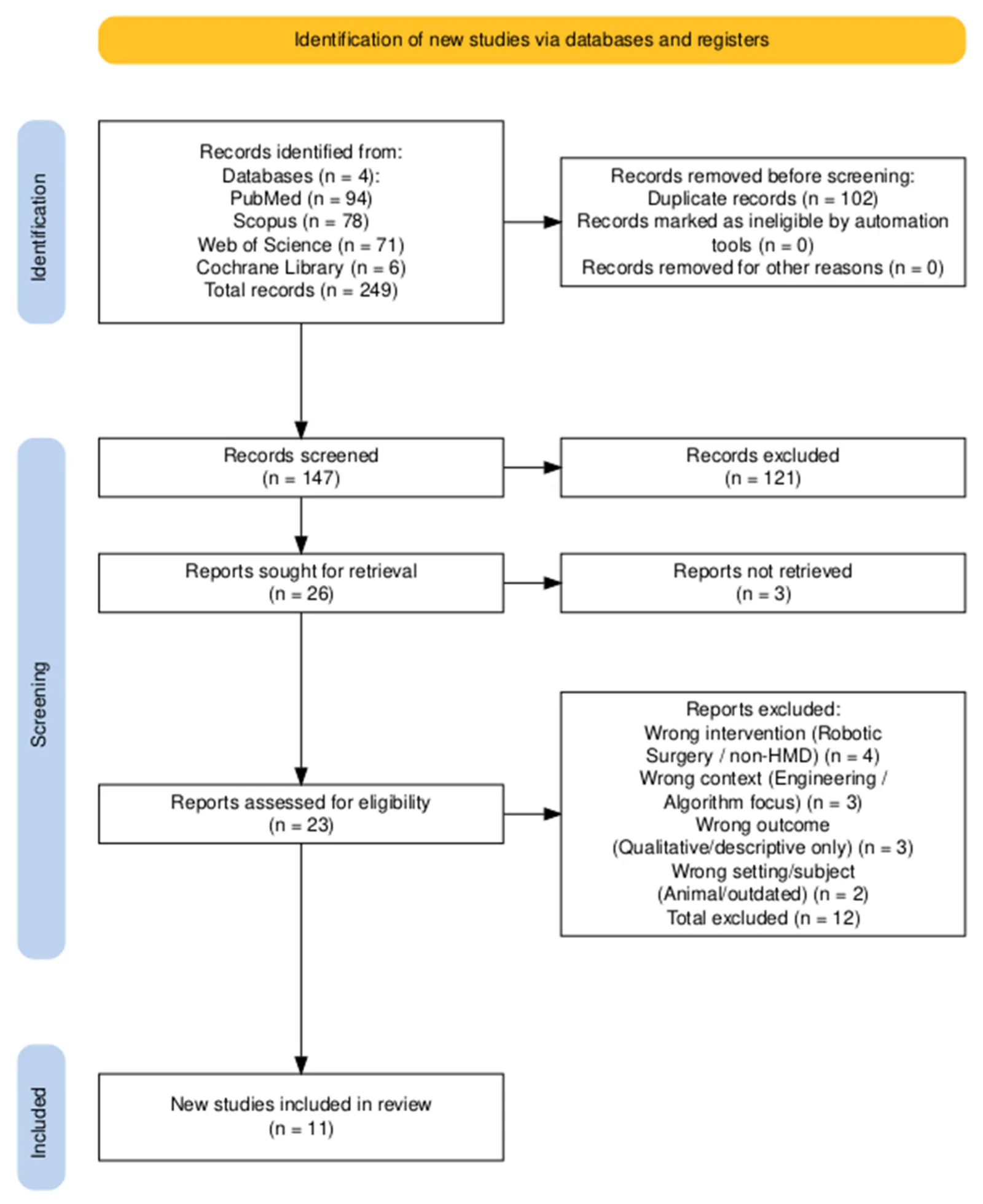
PRISMA-ScR flowchart showing the search and selection process for this review.

Finally, 11 studies met all eligibility criteria and were included in the present scoping review. The selection process is detailed in the PRISMA flow diagram (Figure 1) [31].

### 3.2. Study Characteristics

A total of 11 studies published between 2019 and 2024 were included in this scoping review. Regarding study design, the majority consisted of in vitro investigations (*n* = 7) conducted on phantom heads or 3D-printed models. Clinical evidence is still emerging, with only 3 in vivo clinical studies (including one randomized controlled trial and two case series/reports) and 1 ex vivo cadaver study.

In terms of hardware, the Microsoft HoloLens 2 was the most frequently used Head-Mounted Display (HMD), utilized in 7 out of 11 studies (63.6%). Other devices included the Epson Moverio series (models BT-200 and BT-300), employed in 3 studies (27.3%), and one study utilizing customized monocular AR glasses (9.1%).

Regarding tracking technology, 10 studies utilized optical tracking with physical fiducial markers (90.9%) to maintain registration between the virtual planning and the physical anatomy. These markers ranged from printed patterns (e.g., QR codes, Vuforia patterns) to infrared reflective spheres. Only one study experimented with a markerless tracking algorithm based on dental contour matching (9.1%), highlighting how marker-based systems remain the current standard for precision [23].

### 3.3. Accuracy Assessment (Quantitative Synthesis)

Quantitative data on implant positioning accuracy were extracted and synthesized from the included studies. The analysis reveals a clear distinction between standard implant procedures and complex scenarios (e.g., zygomatic implants) [29,32].

Entry Point Deviation: For standard dental implants, the mean error at the entry point was generally low, ranging from 0.33 ± 0.16 mm to 1.51 ± 0.16 mm. However, in complex scenarios of zygomatic implants, the deviation increased significantly, ranging from 2.43 mm to 5.64 mm.

Apical Deviation: Errors at the implant apex followed a similar trend. In standard cases, apical deviation ranged from 0.19 mm to 1.54 ± 0.14 mm. Conversely, zygomatic implants showed apical deviations ranged from 3.28 ± 2.17 mm to 4.88 mm in comparative in vitro and ex vivo studies.

Angular Deviation: Angular errors varied across studies, ranging from a minimum of 0.85° ± 0.32° to a maximum of 5.56° ± 2.10° for standard implants. In zygomatic cases, the angular deviation reached 9.60° ± 4.25° and a minimum of 5.80° ± 4.12°, indicating the difficulty in maintaining the trajectory over long drilling paths with current AR technology [29,30].

Comparison with Conventional Methods: In comparative studies, AR navigation demonstrated superior accuracy compared to free-hand methods (*p* < 0.05) [24,25,26] in most standard scenarios. When compared to static computer-assisted implant surgery (s-CAIS) or dynamic navigation (DN), AR showed comparable results in linear accuracy but often exhibited slightly inferior angular precision, particularly in full-arch or zygomatic rehabilitations [29,30].

### 3.4. Workflow, Ergonomics, and Technical Barriers

Despite the promising accuracy, several technical and ergonomic barriers have been identified that may limit routine clinical application:

Registration and tracking issues: The most frequently reported technical issue was related to tracking instability. Studies highlighted problems with “jittering” (holographic instability), system latency, and line-of-sight interruptions where the surgeon’s hands or instruments blocked the camera’s view of the markers [19,30,32].

Perceptual and visual issues: Ergonomic concerns were dominated by depth perception errors and visual fatigue. Authors reported the “vergence–accommodation conflict,” where the eyes struggle to simultaneously focus on the virtual overlay (near) and the surgical site (far), causing eye strain [10,19].

Hardware limitations: The weight and bulk of HMDs (particularly HoloLens) were indicated as a cause of cervical spine discomfort during prolonged surgical procedures. Furthermore, limited battery life and hardware intrusiveness in the surgical field were noted as obstacles [29,33,34].

Workflow efficiency: Although some studies reported time efficiency in the drilling phase, the overall workflow was often burdened by a time-consuming setup and calibration phase (approximately 10–30 min) and the need for additional software management compared to conventional analog methods [4,19].

**Table 1:** Included studies data extraction.

Study (Author, Year)	Design	Device	Target/ Scenario	Accuracy (Mean ± SD)	Main Barriers Reported
A. Shusterman et al. 2024 [4]	Case Report (Clinical)	HoloLens 2 (Microsoft Corporation, Redmond, WA, USA)	Single gap	Entry: 0.42 mm Apex: 0.19 mm Angle: 1.85°	Same registration challenges as in vitro; clinical setting adds complexity.
S. Arunjaroensuk et al. 2024 [10]	RCT (Clinical)	Epson Moverio BT-300 (Seiko Epson Corporation, Suwa, Japan)	Single gap	Entry: 0.75 ± 0.45 mm Apex: 0.87 ± 0.45 mm Angle: 1.47 ± 1.01°	Expense of AR technology, setup time, additional software required. Wireless connection lag and battery degradation. Uncomfortable virtual screen positioning requiring awkward head tilting.
G. Pellegrino et al. 2019 [19]	Case Report (Clinical)	Epson Moverio BT-200 (Seiko Epson Corporation, Suwa, Japan)	Single gap	Entry: 0.53 and 0.46 mm Apex: 0.50 and 0.48 mm Angle: 3.05 and 2.19°	System lag, hologram instability (jitter), depth perception issues. Focal distance accommodation required.
X. Fan et al. 2023 [20]	In Vitro	HoloLens 2 (Microsoft Corporation, Redmond, WA, USA)	Partial edentulism	Entry: 1.51 ± 0.16 mm Apex: 1.54 ± 0.14 mm Angle: 3.47 ± 0.34°	None specifically reported (Validation study focused on accuracy).
S. Dong et al. 2023 [23]	In Vitro	Custom AR Glasses	Partial edentulism	Entry: 1.17 ± 0.24 mm Apex: 1.43 ± 0.39 mm Angle: 5.56 ± 2.10°	Latency dependent on lighting conditions (markerless tracking).
A. Shusterman et al. 2024 [24]	In Vitro	HoloLens 2 (Microsoft Corporation, Redmond, WA, USA)	Partial edentulism	Entry: 0.33 ± 0.16 mm Apex: 0.40 ± 0.16 mm Angle: 0.85 ± 0.32°	Registration phase requires “full concentration” and stable environment.
J.R. Gonzalez-Rueda et al. 2023 [29]	In Vitro	HoloLens 2 (Microsoft Corporation, Redmond, WA, USA)	Zygomatic	Entry: 5.64 ± 1.11 mm Apex: 4.88 ± 1.54 mm Angle: 9.60 ± 4.25°	Device weight causing physical fatigue. Visual fatigue (Vergence–Accomodation Conflict), limited battery life.
S.T. Heijtmeijer et al. 2024 [30]	Cadaver (Ex Vivo)	HoloLens 2 (Microsoft Corporation, Redmond, WA, USA)	Zygomatic	Entry: 2.43 ± 1.33 mm Apex: 3.28 ± 2.17 mm Angle: 5.80 ± 4.12°	Line-of-sight issues, depth perception errors. Headset weight causing discomfort.
M. Kivovics et al. 2022 [32]	In Vitro	Epson Moverio BT-300 (Seiko Epson Corporation, Suwa, Japan)	Partial edentulism	Entry: 1.27 ± 0.40 mm Apex: 1.34 ± 0.41 mm Angle: 4.09 ± 2.79°	Line-of-sight interruption (hands blocking markers). Smart glasses are lightweight but have limited Field of View (FOV).
L. Liu et al. 2023 [33]	In Vitro	HoloLens 2 (Microsoft Corporation, Redmond, WA, USA)	Partial edentulism	Entry: 0.69 ± 0.25 mm Apex: 0.79 ± 0.23 mm Angle: 1.85 ± 0.61°	Battery life limits long procedures. Visual fatigue from prolonged use. Device weight.
B. Tao et al. 2024 [34]	In Vitro	HoloLens 2 (Microsoft Corporation, Redmond, WA, USA)	Total edentulism	Entry: 1.31 ± 0.67 mm Apex: 1.36 ± 0.67 mm Angle: 3.72 ± 2.13°	Visualization scale (1:1 projection hides small errors). Headset weight/bulk causing cervical spine issues.

## 4. Discussion

### 4.1. Synthesis of Evidence

This scoping review has mapped the state of the art of dynamic navigation via Augmented Reality (AR) supported by wearable devices (Head-Mounted Displays, HMDs) in implant dentistry, focusing specifically on studies where the technology was actively utilized in in vitro, ex vivo, or in vivo settings. The analysis of the 11 included studies (published between 2019 and 2024) outlines a technological landscape in a critical transitional phase: while accuracy in standard cases is promising, significant ergonomic and technical barriers persist, limiting its routine clinical use [10,19,24]. Current evidence suggests that AR navigation reaches accuracy values that perform significantly better than free-hand methods under experimental conditions, and devices such as the Microsoft HoloLens 2 and Epson Moverio have demonstrated the ability to guide the surgeon with a clinically acceptable margin of error. However, it must be strongly emphasized that the majority of these findings derive from laboratory models (in vitro) or cadavers (ex vivo). The few available in vivo studies involve very small patient cohorts, which currently prevents the generalization of these results and their direct translation to standard clinical scenarios. Furthermore, when compared to static computer-assisted implant surgery (s-CAIS), it is crucial to note that the control groups in the evaluated literature often utilized tooth-supported templates [24]. These physical guides inherently provide a level of stability and precision that current HMD-based AR cannot consistently match. Finally, the review highlights a clear performance dichotomy between standard rehabilitations and complex scenarios, such as zygomatic implants. While comparing a standard implant to a 30–50 mm zygomatic implant is fundamentally asymmetrical—due to the massive amplification of apical deviation caused by the lever arm effect—this comparison is reported in the present Scoping Review to demonstrate that AR technology is actively being investigated for advanced oral and maxillofacial implant surgeries. Although preliminary results in these complex cases are encouraging, the system’s susceptibility to tracking errors under conditions of poor marker visibility confirms that wearable AR is still far from clinical routine, indicating that its Technology Readiness Level is not yet uniform across all surgical indications [24,29,32].

It should be explicitly noted that the quantitative data and the clinical comparisons discussed herein—namely, AR navigation versus s-CAIS, d-CAIS, and free-hand methods, as well as the juxtaposition of standard and zygomatic implants—must not be interpreted as a formal statistical meta-analysis. In alignment with the fundamental purpose of a scoping review, these findings are reported strictly to provide the reader with a comprehensive map of the current bibliographic landscape, illustrating the emerging trends and outlining the current boundaries of this technology in dental implantology.

### 4.2. Accuracy Analysis: The “Gap” Between Standard and Complex

The scientific literature presents diverse findings regarding the accuracy of Augmented Reality-based Dynamic Navigation (AR-DN) for dental implant placement, largely due to the rapid evolution of the technology and the variety of systems tested. While many studies support the superiority of AR-DN over traditional methods, the evidence remains somewhat heterogeneous [24,32].

Several studies indicate that AR-DN significantly outperforms conventional Freehand (FH) techniques and offers precision comparable to Static Computer-Assisted Implant Surgery (s-CAIS):

Kivovics et al. (2022) [32]: In an in vitro study utilizing 3D-printed models, researchers found no significant difference in accuracy between AR-DN and s-CAIS. However, both guided methods demonstrated significantly smaller coronal, apical, and angular deviations compared to the FH approach [32].

Liu et al. [33]: This independent in vitro investigation corroborated the advantages of AR-DN over FH placement, reporting enhanced precision in entry point, apical depth, and angular trajectory [33].

Dong et al. [23]: By testing a markerless AR guidance system, this research group demonstrated that the technological deviations in the neck, apex, and angulation of the implants successfully met required clinical standards [23].

Despite the promising outcomes highlighted above, some investigations suggest that AR-DN may not yet be universally superior in all surgical scenarios:

Tao et al. [34]: When directly comparing AR-DN to conventional Dynamic Navigation (DN) in an in vitro setup, researchers found that while coronal and apical deviations were similar between the two groups, conventional DN allowed for significantly smaller angular deviations than the AR-based system [34].

Gonzalez-Rueda et al. [29]: In a study evaluating the highly complex placement of zygomatic implants in polyurethane models, the authors reported contrary results. Their findings indicated that the traditional FH method was more accurate than all the computer-assisted surgical techniques evaluated—including s-CAIS, conventional DN, and AR-DN—particularly concerning apical and angular deviations [29].

One of the most relevant findings of this review is the variability of accuracy depending on anatomical complexity. In scenarios of partial or single edentulism, AR showed mean entry point deviations ranging between 0.33 mm and 1.51 mm and mean apex deviations ranging between 0.19 mm and 1.54 mm. These values are comparable to those reported in the literature for static computer-assisted implant surgery (s-CAIS) and superior to the free-hand technique. Studies such as those by Shusterman et al. and Fan et al. confirm that, under controlled conditions, holographic superimposition is sufficiently precise to ensure the safety of vital anatomical structures in the posterior regions [20,24].

However, critical data analysis reveals a major issue in the management of zygomatic implants. Studies by Gonzalez-Rueda et al. and Heijtmeijer et al. report mean angular deviations up to 9.60° and apical deviations up to 4.88 mm. This phenomenon can be explained by the “lever arm” effect: on extra-long implants (30–50 mm), even a millimeter error in sensor tracking at the entry point translates into a massive deviation at the apex. Unlike surgical templates, which offer a physical constraint along the entire drilling path, AR provides only visual guidance; in the absence of haptic feedback or a robotic arm, maintaining the trajectory over long distances remains an open challenge for the human hand guided solely by holograms [23,29,30]. The discrepancies observed across these studies can be primarily attributed to differences in the specific AR headsets utilized, the proprietary software algorithms driving the holograms, and the inherent computational capabilities of the testing devices. While AR-DN shows immense potential as a highly accurate alternative to FH and s-CAIS in standard implantology, its efficacy in complex anatomical scenarios requires further technological refinement and validation [23,24,32]. Given that these findings remain limited to a small cohort of studies—predominantly in a pre-clinical phase—it is important to emphasize that the primary aim of this scoping review is to map the current state of accuracy and ergonomic barriers associated with AR-based implant navigation.

### 4.3. Technical and Ergonomic Barriers

Despite the promising accuracy, the review identified three macro-areas of critical issues that hinder the routine clinical implementation of the technology:

1. Tracking Instability and “Line-of-Sight”: Most of the included studies (10 out of 11) rely on optical tracking systems with physical markers (reflective spheres or QR/Vuforia patterns). An intrinsic limitation frequently reported, for example in Kivovics et al., is the interruption of the line of sight (Line-of-Sight occlusion): the surgeon’s hands, the assistant, or the instrument itself can obscure the markers, causing the sudden disappearance of the guiding hologram or “jitter” phenomena (flickering of the virtual image) [19,32]. This forces the operator to interrupt the surgical workflow to restore tracking, increasing intraoperative stress.
2. Hardware Limitations and Cervical Discomfort: The predominant device, HoloLens 2, although high-performing in terms of spatial computing, has been criticized for its weight and bulk. Studies such as Tao et al. and Heijtmeijer et al. report fatigue of the cervical muscles during lengthy procedures [30,34]. The alternative represented by lighter Smart Glasses (e.g., Epson Moverio, used by Arunjaroensuk and Pellegrino) solves the weight issue but introduces limitations in the Field of View (FOV) and often requires a cable connection (tethering) that reduces freedom of movement [10,19].
3. Vergence–Accommodation Conflict (VAC) and Depth Perception: A subtle but critical perceptual issue is the vergence–accommodation conflict. The surgeon’s eye must accommodate (focus on) the virtual hologram projected at a fixed distance (usually 1–2 optical meters) while converging their gaze on the real surgical site located 30–40 cm away. Gonzalez-Rueda et al. and Pellegrino et al. explicitly reported issues of visual fatigue and altered depth perception, which can lead to over-preparation errors or incorrect vertical positioning of the implant [19,29].

### 4.4. Workflow and Learning Curve

The integration of AR into the digital workflow does not come at a “zero cost”. Although the actual drilling time can be reduced thanks to direct egocentric visualization (which allows the surgeon to maintain focus on the surgical field without looking away at external monitors), the overall chair time is often increased by setup procedures. Arunjaroensuk et al., in the only included clinical RCT, highlighted how the initial calibration and marker registration require additional time and a non-negligible learning curve for the surgical team [10]. Currently, the logistical complexity of the AR setup does not yet outweigh the perceived benefits for routine and simple clinical cases.

### 4.5. Study Limitations

The results of this scoping review must be interpreted considering some limitations. Firstly, the preponderance of in vitro studies (7 out of 11) represents a significant bias. A formal Risk of Bias was not performed; however, it is prudent to take into consideration the heterogeneity of the studies considered, the relatively low number of them and the different experimental settings; Technical and methodological limitations to be considered:

Resin models do not replicate the challenges of the real oral environment: the presence of saliva, blood, lens fogging, involuntary patient movements, and limited mouth opening. Therefore, it is likely that the reported accuracy data are overestimated compared to clinical reality. Secondly, the heterogeneity of error measurement protocols (some studies use pre/post CBCT superimpositions, while others use direct analyses) makes a direct comparison or formal meta-analysis difficult.

### 4.6. Future Perspectives

To bridge the gap between research and clinical practice, future development must focus on:

- Markerless Tracking: The elimination of physical markers, as preliminarily attempted by Dong et al. using dental contour recognition, is the key to solving bulk and line-of-sight issues [23].
- Advanced Hardware: The adoption of next-generation “waveguide” optics could reduce the weight of the glasses and mitigate the vergence–accommodation conflict [10,23].
- AI Integration: Artificial intelligence algorithms could compensate for patient movements in real-time, making tracking robust even under dynamic conditions [23].
- Software Improvements: Touchless interfaces; matching of the virtual and real anatomy of the patient; holographic delay, imprecision of the superimposition and haptic feedback should be implemented [19,24,32,33].
- Long-term Clinical Studies: Across all included studies, there is a consensus among authors regarding the necessity for rigorous, long-term clinical trials to assess the accuracy, safety, and reproducibility of this auxiliary technology in the field of implant dentistry.

## 5. Conclusions

This scoping review aimed to map the existing literature on the application of wearable AR devices in implant dentistry. This objective has been fulfilled, providing a clear overview of current research trends, the technological state of the art, and the key points upon which future investigations should concentrate.

While the preliminary results demonstrate the interesting potential of holographic navigation during implant placement, the technology is currently in a predominantly preclinical phase. Since most of the available evidence is based on in vitro studies utilizing resin models/phantoms, these findings do not accurately replicate the complexities of a true surgical environment, such as limited mouth opening, soft tissue mobility, and tracking instability caused by blood or saliva. Consequently, the clinical evidence remains too limited to justify the routine implementation of AR HMDs in daily practice, and current data are insufficient to claim that these systems can consistently match the reliability of established static computer-assisted implant surgery (s-CAIS) or dynamic computer-assisted implant surgery (d-CAIS). Furthermore, significant technical and ergonomic barriers persist. Issues such as jittering (tracking instability), hardware weight, and the vergence–accommodation conflict currently hinder the system’s viability, particularly in complex or lengthy rehabilitations like zygomatic implant placement. Future research must prioritize the transition from laboratory settings to rigorous, long-term in vivo clinical trials. Technologically, further hardware miniaturization and the evolution of robust markerless tracking software are strictly required before wearable AR can be considered a safe and standard tool for dental implant surgery.

## Data Availability

Data availability can be retrieved by the following databases with the following prompts: Pubmed: (“Augmented Reality”[Mesh] OR “Mixed Reality”[Title/Abstract] OR “Extended Reality”[Title/Abstract] OR “AR”[Title/Abstract] OR “HoloLens”[Title/Abstract] OR “MR”[Title/Abstract] OR “Apple Vision Pro”[Title/Abstract] OR “Meta Quest”[Title/Abstract] OR “Head-mounted display”[Title/Abstract] OR “Smart glasses”[Title/Abstract] OR “Augmented Reality-assist*”[Title/Abstract]) AND (“Dental Implants”[Mesh] OR “Implantology”[Title/Abstract] OR “Implant placement”[Title/Abstract] OR “Dental implant*”[Title/Abstract] OR “Endosseous”[Title/Abstract] OR “Guided implant surgery”[Title/Abstract] OR “Atrophic Maxilla”[Mesh] OR “Dental Implantation, Endosseous”[Mesh]) AND (“Accuracy”[Title/Abstract] OR “Deviation”[Title/Abstract] OR “Registration Error”[Title/Abstract] OR “Workflow”[Title/Abstract] OR “Precision”[Title/Abstract] OR “Ergonomics”[Title/Abstract] OR “Time”[Title/Abstract]) NOT (“Endodontic”[Title/Abstract] OR “Orthognathic”[Title/Abstract] OR “Tumor”[Title/Abstract] OR “Education”[Title/Abstract] OR “Training”[Title/Abstract] OR “Students”[Title/Abstract]). Scopus: TITLE-ABS-KEY ((“Augmented Reality” OR “Mixed Reality” OR “Extended Reality” OR “HoloLens” OR “Apple Vision Pro” OR “Meta Quest” OR “Head-mounted display” OR “Smart glasses”)) AND TITLE-ABS-KEY ((“Dental Implants” OR “Implantology” OR “Implant placement” OR “Dental implant*” OR “Endosseous” OR “Guided implant surgery” OR “Atrophic Maxilla” OR “Dental Implantation”)) AND TITLE-ABS-KEY ((“Accuracy” OR “Deviation” OR “Registration Error” OR “Workflow” OR “Precision” OR “Ergonomics” OR “Time”)) AND NOT TITLE-ABS-KEY ((“Endodontic” OR “Orthognathic” OR “Tumor” OR “Education” OR “Training” OR “Students”)). Web of Science (WoS): TS = ((“Augmented Reality” OR “Mixed Reality” OR “Extended Reality” OR “HoloLens” OR “Apple Vision Pro” OR “Meta Quest” OR “Head-mounted display” OR “Smart glasses”) AND (“Dental Implants” OR “Implantology” OR “Implant placement” OR “Dental implant*” OR “Endosseous” OR “Guided implant surgery” OR “Atrophic Maxilla” OR “Dental Implantation”) AND (“Accuracy” OR “Deviation” OR “Registration Error” OR “Workflow” OR “Precision” OR “Ergonomics” OR “Time”)) NOT TS = (“Endodontic” OR “Orthognathic” OR “Tumor” OR “Education” OR “Training” OR “Students”). Cochrane Library: (“Augmented Reality” OR “Mixed Reality” OR “Extended Reality” OR “HoloLens” OR “Apple Vision Pro” OR “Meta Quest” OR “Head-mounted display” OR “Smart glasses”) AND (“Dental Implants” OR “Implantology” OR “Implant placement” OR “Dental implant*” OR “Endosseous” OR “Guided implant surgery” OR “Atrophic Maxilla” OR “Dental Implantation”) AND (“Accuracy” OR “Deviation” OR “Registration Error” OR “Workflow” OR “Precision” OR “Ergonomics” OR “Time”) NOT (“Education” OR “Training” OR “Students”).

## Abbreviations

The following abbreviations are used in this manuscript:

AI	Artificial Intelligence
AR	Augmented Reality
AV	Augmented Virtuality
CAD	Computer Aided Design
CAIS	Computer-Assisted Implant Surgery
CBCT	Cone Beam Computed Tomography
d-CAIS	Dynamic Computer-Assisted Implant Surgery
DICOM	Digital Imaging and Communications in Medicine
DN	Dynamic Navigation
HMD	Head Mounted Display
IOS	Intra Oral Scanner
JBI	Joanna Briggs Institute
MeSH	Medical Subject Headings
MR	Mixed Reality
OMS	Oral and Maxillofacial Surgery
PCC	Population, Concept, Context
PRISMA-ScR	Preferred Reporting Items for Systematic reviews and Meta-Analyses extension for Scoping Reviews
RCT	Randomized Controlled Trial
s-CAIS	Static Computer-Assisted Implant Surgery
SD	Standard Deviation
STL	Standard Tessellation Language
VR	Virtual Reality
